# Supplementary material for: Systemic acquired resistance networks amplify airborne defense cues
Source: Nat Commun. 2019 Aug 23;10:3813. doi: 10.1038/s41467-019-11798-2 (PMC6707303; doi:10.1038/s41467-019-11798-2)
Supplement: Supplementary file 1 — Supplementary Information [file 41467_2019_11798_MOESM1_ESM.pdf]

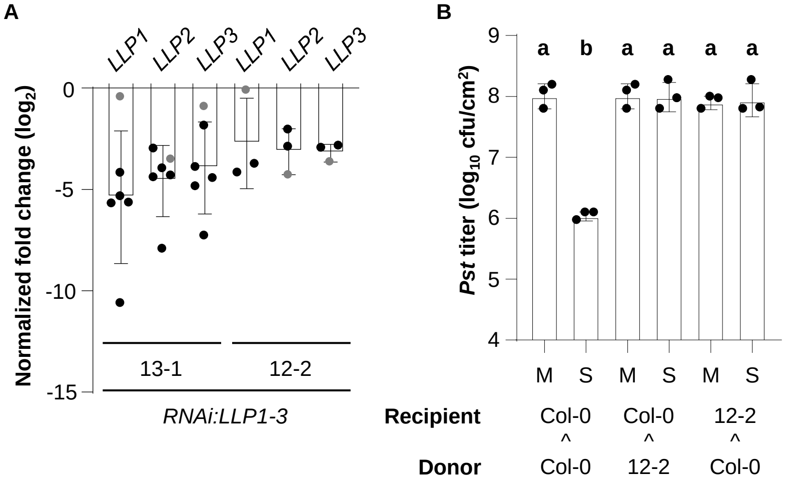

Supplementary Figure 1 *LLP1*, *LLP2*, and *LLP3* transcript accumulation in *RNAi:LLP1-3* and their local and systemic contributions to systemic acquired resistance (SAR). (A) *LLP1*, *LLP2*, and *LLP3* transcript accumulation in *RNAi:LLP1-3* plants of two independent transgenic lines (12-2 and 13-1) relative to that in Col-0 wild type plants. *LLP1*, *LLP2*, and *LLP3* transcript levels were normalized to *UBIQUITIN* in each sample prior to further analysis. Genes are indicated above bars and plant genotypes below the bars. Dots indicate individual results from biologically independent experiments (in black: data derived from leaves of 4-5-week-old plants; in grey: data derived from seedlings; n=3 (12-2) to 6 (13-1)). Bars represent the average of the indicated results ± standard deviation. (B) Petiole exudate experiment as in Fig. 1. Leaves of donor plants were inoculated with *Pst/AvrRpm1* (SAR-induced; S) or mock-treated (M). 24 h later, their petiole exudates were collected and infiltrated into the leaves of naïve recipient plants. The same leaves were inoculated with *Pst* and the resulting *in planta Pst* titers are shown at 4 dpi. The treatments of the donor plants are indicated below the bars. The donor and recipient genotypes are indicated below the panels. Dots indicate 3 replicates from 1 experiment ± standard deviation. Different letters above bars indicate significant differences, one-way ANOVA, $P<0.05$. Abbreviations: *LLP1*, *LEGUME LECTIN-LIKE PROTEIN 1*; *Pst*, *Pseudomonas syringae* pathovar *tomato*; cfu, colony-forming units

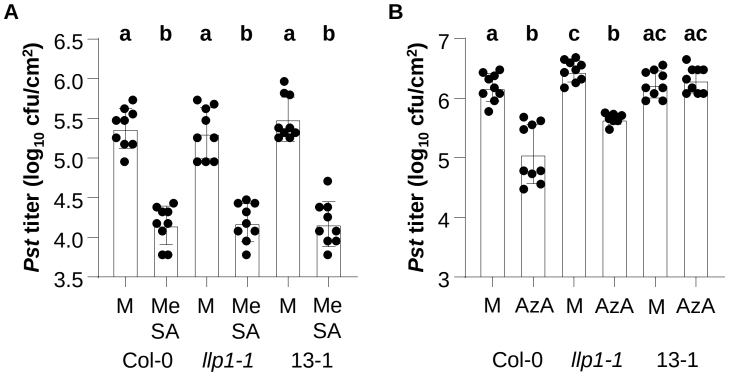

Supplementary Figure 2 LLP1, 2, and/or 3 contribute to azelaic acid (AzA)-, but not methyl salicylate (MeSA)-induced immunity. (A) MeSA-induced resistance. Plants were exposed to MeSA in air-tight containers or to the solvent hexane as the mock (M) control treatment as indicated below the panel. The plants were subsequently released from the containers and inoculated in the leaves with *Pst.* The resulting *in planta Pst* titers at 4 dpi are shown. (B) AzA-induced resistance. Plants were infiltrated in their first and second true leaves with AzA. Systemic leaves were subsequently inoculated with *Pst*, and the resulting *in planta Pst* titers at 4 dpi are shown. (A/B) Plant genotypes are indicated below the panels. Dots indicate individual results from 3 biologically independent experiments per genotype and treatment (including 3 replicates from each experiment). Bars represent the average of the indicated results ± standard deviation. Different letters above bars indicate significant differences, one-way ANOVA, P<0.05. Grubb's outlier test identified 1 statistically significant outlier in the data set *llp1-1* AzA-treated; this outlier was excluded from further analyses to assure normal distribution of the remaining data and is highlighted in grey in the source data file associated with this manuscript.

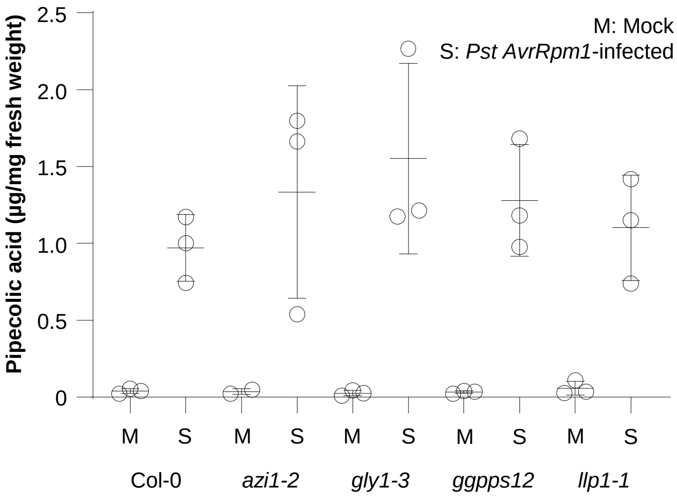

Supplementary Figure 3 Infection-induced pipecolic acid levels are normal in *azi1-2*, *gly1-3*, *ggpps12*, and *llp1-1* plants. Pipecolic acid accumulation is shown in leaves of the plant genotypes indicated below the panel 3 d after inoculation of the leaves with *Pst/AvrRpm1* (S) or the corresponding mock (M) treatment. Circles indicate results from 3 biologically independent measurements. Lines show the average of the indicated results ± standard deviation. The data were analyzed using one-way ANOVA, and no significant differences were observed.

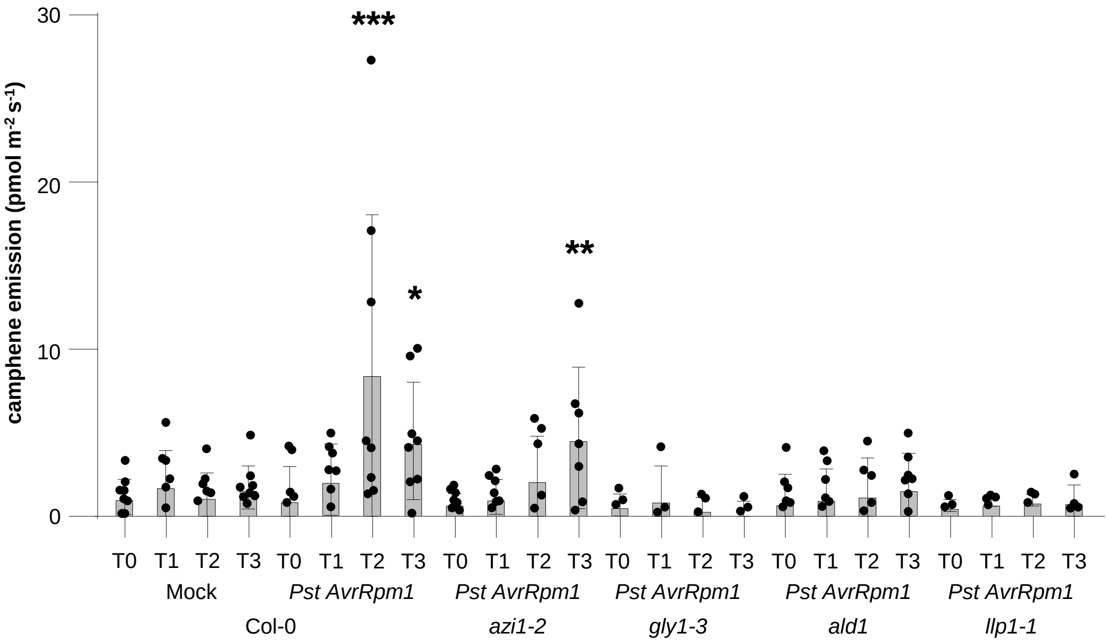

Supplementary Figure 4 Camphene emission rates from different plant genotypes. Genotypes are indicated below the panel and camphene emissions were monitored 1 d before (T0) and during the first (T1), second (T2, and third (T3) d after spray inoculation of the plants with *Pst/AvrRpm1* or the corresponding mock treatment. Dots indicate individual, biologically independent results (n=3-8); bars represent the average of the indicated results ± standard deviation. Asterisks indicate significant differences from the respective Col-0 mock control. Genotype main effect: Col-0***. Two-way ANOVA, multiple comparison versus control groups, Holm-Sidak method; * P<0.05, ** P<0.01, *** P<0.001.

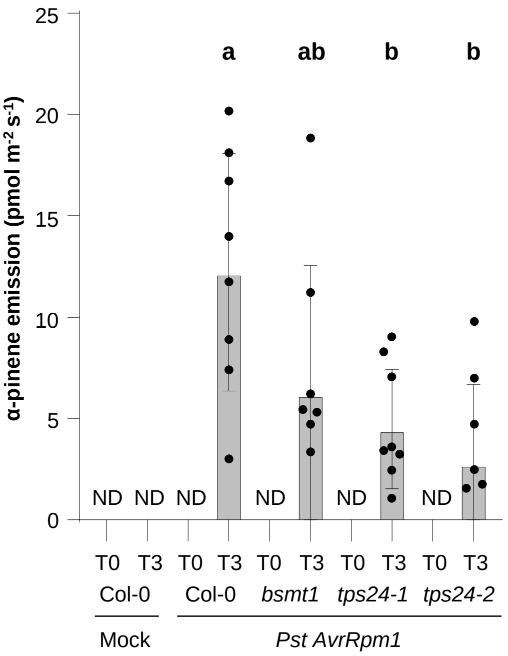

Supplementary Figure 5 α-Pinene emission rates from *bsmt1* and *tps24* plants. α-Pinene emission rates are shown of the plant genotypes indicated below the panel one day (d) before (T0) and during the third d (T3) after spray inoculation with *Pst/AvrRpm1* or the corresponding mock treatment. Dots indicate individual, biologically independent results (n=6-8); bars represent the average ± standard deviation. Different letters above bars indicate significant differences, one-way ANOVA, $P<0.05$. ND: not detectable indicates that the average emission rates remained below background levels. Individual data that were below background levels are not indicated in the bars, but were included in statistical analyses of the data and in the source data file associated with this manuscript. Grubb's outlier test identified 1 statistically significant outlier in the data set *tps24-1* T0; this outlier was excluded from further analyses to assure normal distribution of the remaining data and is highlighted in grey in the source data file.

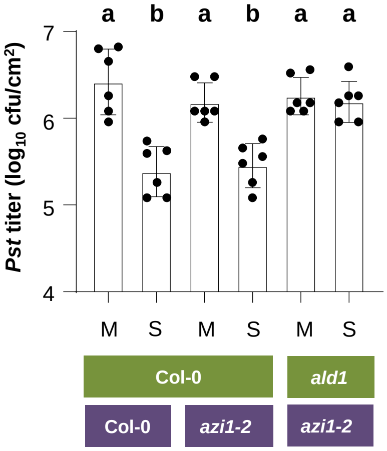

Supplementary Figure 6 Plant-to-Plant (PTP) experiment between *azi1-2* and *ald1.* Sender plants were either inoculated with *Pst/AvrRpm1* (SAR-induced; S) or mock-treated (M), and incubated with naïve receiver plants in airtight containers. The receivers were subsequently released from the containers and inoculated with *Pst.* The resulting *in planta Pst* titers at 4 dpi of the receivers are shown. Plant genotypes are indicated below the panels (senders in purple and receivers in green). The treatments of the senders (M or S) are indicated below the bars. Dots indicate individual results from 2 biologically independent experiments, including 3 replicates each. Bars represent the average of the indicated results ± standard deviation. Different letters above bars indicate significant differences, one-way ANOVA, P<0.05.

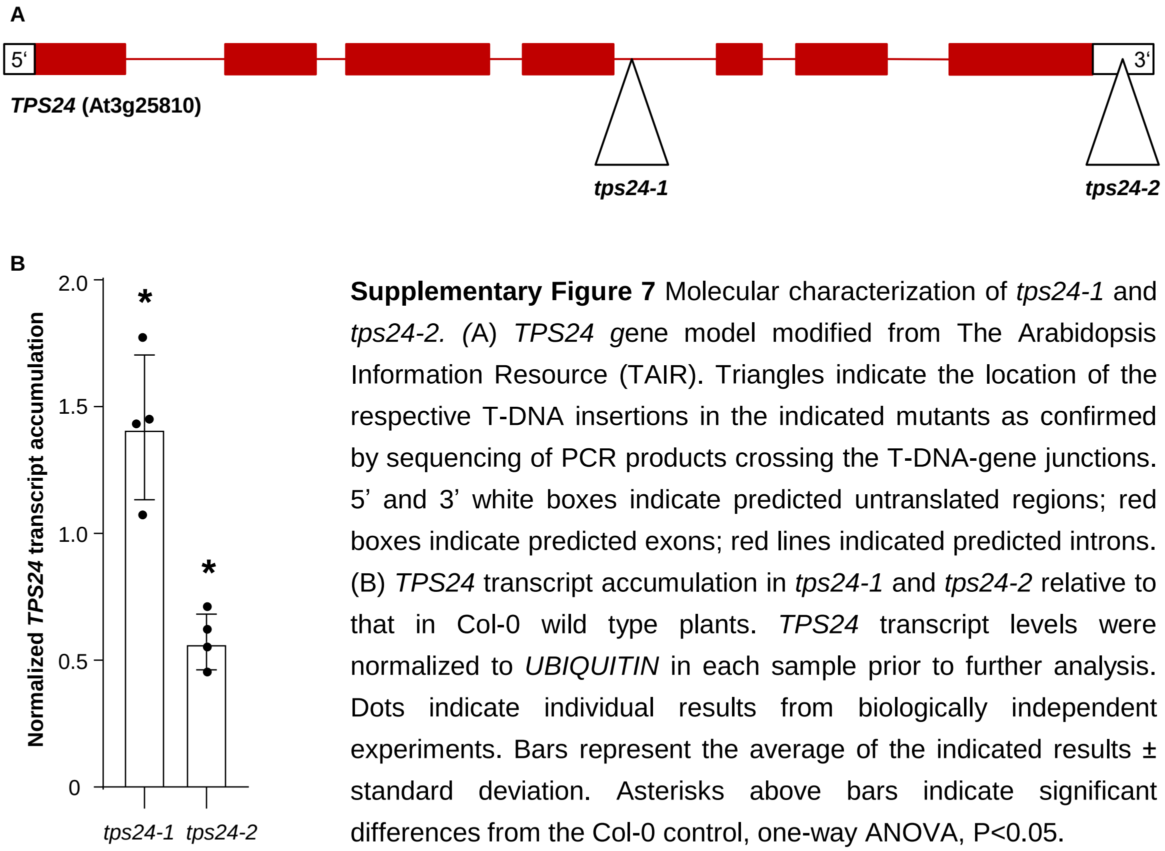

Supplementary Figure 7 Molecular characterization of *tps24-1* and *tps24-2. (*A) *TPS24 g*ene model modified from The Arabidopsis Information Resource (TAIR). Triangles indicate the location of the respective T-DNA insertions in the indicated mutants as confirmed by sequencing of PCR products crossing the T-DNA-gene junctions. 5' and 3' white boxes indicate predicted untranslated regions; red boxes indicate predicted exons; red lines indicated predicted introns. (B) *TPS24* transcript accumulation in *tps24-1* and *tps24-2* relative to that in Col-0 wild type plants. *TPS24* transcript levels were normalized to *UBIQUITIN* in each sample prior to further analysis. Dots indicate individual results from biologically independent experiments. Bars represent the average of the indicated results ± standard deviation. Asterisks above bars indicate significant differences from the Col-0 control, one-way ANOVA, $P<0.05$.

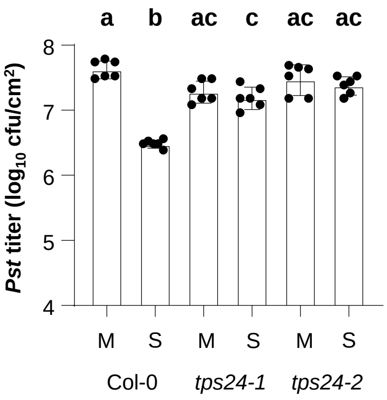

Supplementary Figure 8 SAR is abolished in *tps24* mutants. SAR was induced in the genotypes indicated below the panel by a local infiltration of the first two true leaves of the plants with *Pst/AvrRpm1* (S). For comparison, a separate set of plants was treated with a mock solution in the same leaves (M). Three d later, systemic leaves were inoculated with *Pst*, and the resulting *in planta Pst* titers at 4 dpi are shown. Dots indicate individual results from 2 biologically independent experiments, including 3 replicates each. Bars represent the average of the indicated results ± standard deviation. Different letters above bars indicate significant differences, one-way ANOVA, $P<0.05$.

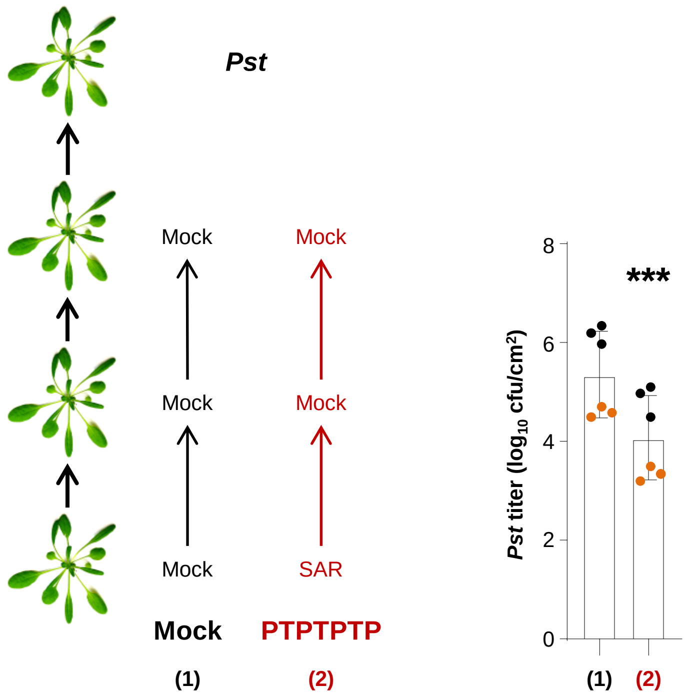

Supplementary Figure 9 Plant-to-plant-to-plant-to-plant (PTPTPTP) experiment. This experiment was performed in essentially the same way as the experiment shown in Fig. 6. In 3 successive rounds of co-incubations, *Pst/AvrRpm1*-infected (SAR-induced) and mock-treated sender plants were incubated with untreated receiver plants that became the senders in the next round. Each co-incubation of sender and receiver plants was performed in an air-tight container that was opened once per day to let in fresh air and exchanged for a clean container between co-incubation rounds. The treatment series 1 and 2 are shown on the left and indicated below the panel on the right. The third receiver was inoculated with *Pst* and the resulting *in planta Pst* titers at 4 dpi are shown (on right). Dots indicate individual results from 2 biologically independent experiments (indicated by different colors), including 3 replicates each. Bars represent the average of the indicated results ± standard deviation. Asterisks above bar indicate a significant difference from the mock control (paired *t* test, P<0.001).

Supplementary Table 1 Oligonucleotides used in this study

**Primer name**	**Sequence (5' to 3')**
Primers for genotyping	
pLBb1.3	ATTTTGCCGATTTCGGAAC
tps24-1-R	TGTGTTTAATATATCCCATATTCACG
tps24-2-R	TTCTAAAACCCTGGCCTTTTG
Primers for generating RNAi constructs	
LLP3-RNAi-F	CACCCTCGAGGGATCCACTTCGATTCCTTCGATGGC
LLP3-RNAi-R	CCGCCATCTGAAGCACCGGGGCTGGGACAATGACGAAG
LLP2-RNAi-F	CTTCGTCATTGTCCCAGCCCCGGTGCTTCAGATGGCGG
LLP2-RNAi-R	CGGCGGAAGTGGTGTGAGCTTCAGGCGCGAGTGTAACCG
LLP1-RNAi-F	CGGTTACACTCGCGCCTGAAGCTCACACCACTTCCGCCG
LLP1-RNAi-R	GGTACCAAGCTTAAGCAAGGCCGTGACCAGGG
Primers for qPCR	
LLP3-fwd	TTTGGAGCTGGTCGTTTG
LLP3-rev	ATTCACTCTACAACAATT
LLP2-fwd	CCCGAGACGAGAACCCAT
LLP2-rev	GGAGTTATCAGCTTCCGGGG
TPS24-fwd	TGCAATGAACCTGGCACGTA
TPS24-rev	CGATCTTGGCTTTGTCGGGA
